# How to define a dolphin “group”? Need for consistency and justification based on objective criteria

**DOI:** 10.1002/ece3.9513

**Published:** 2022-11-18

**Authors:** Jonathan Syme, Jeremy J. Kiszka, Guido J. Parra

**Affiliations:** ^1^ Cetacean Ecology, Behaviour and Evolution Lab, College of Science and Engineering Flinders University Adelaide South Australia Australia; ^2^ Institute of Environment, Department of Biological Sciences Florida International University North Miami Florida USA

**Keywords:** behavioral ecology, Cetacea, Delphinidae, group dynamics, grouping behavior, marine mammals

## Abstract

Group living is a critical component of the ecology of social animals such as delphinids. In many studies on these animals, groups represent sampling units that form the basis of the collection and analysis of data on their abundance, behavior, and social structure. Nevertheless, defining what constitutes a group has proven problematic. There is inconsistency in the terms and criteria used and many definitions lack biological justification. We conducted a literature review and an online expert survey to assess various terms (*group*, *school*, *party*, and *pod*), and their definitions as applied to delphinids to identify issues to ultimately make recommendations. Of 707 studies analyzed, 325 explicitly defined one or more terms, providing 344 definitions. Additionally, 192 definitions were obtained from the survey. Among these definitions, *group* was the most common term used (review: 286 definitions, 83.1%; survey: 69 definitions, 35.9%) and the most familiar to the survey respondents (73 respondents, 100.0%). In definitions of *group*, spatial proximity was the most used criterion (review: 200 definitions, 71.2%; survey: 53 definitions, 81.5%) followed by behavior (review: 176 definitions, 62.6%; survey: 38 definitions, 58.5%). The terms and criteria used to define delphinid groups vary considerably. Rather than proposing a single formal definition, we instead recommend that the term *group* and spatial proximity criteria be used to define sampling units of individuals observed in the field. Furthermore, we propose a process for formulating definitions that involves analyzing interindividual distances to determine naturally occurring patterns that are indicative of group membership. Although this process is based principally on the spatial proximity of individuals, it could also incorporate the behavior of group members by evaluating the influence of behavior on interindividual distances. Such a process produces definitions that are biologically meaningful and compatible across studies and populations, thus increasing our ability to draw strong conclusions about group living in delphinids.

## Graphical abstract

Group living is a critical component of the ecology of social animals such as delphinids, yet defining what constitutes a group has proven problematic. We conducted a literature review and an online expert survey to assess various terms and their definitions as applied to delphinids to identify issues to ultimately make recommendations. We recommend that the term group and spatial proximity criteria be used to define sampling units of individuals observed in the field and we propose a process for formulating biologically meaningful definitions.

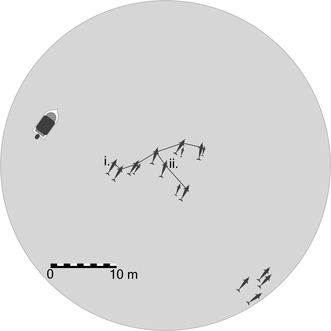

## 1 INTRODUCTION

Animal groups consist of interacting individuals that actively achieve and maintain spatial proximity due to attraction between them (Connor, 2000; Krause & Ruxton, 2002; Majolo & Huang, 2018; Whitehead, 2008). As such, groups differ from aggregations of individuals that form due to an external factor (e.g., a food resource) (Croft et al., 2008; Krause & Ruxton, 2002; Whitehead, 2008). The attraction between grouped individuals stems from the evolutionary basis for group living—the benefits gained from grouping (e.g., reduced predation risk, improved foraging, and enhanced reproductive opportunities) outweigh the costs (e.g., increased interindividual competition and disease transmission) (Krause & Ruxton, 2002; Majolo & Huang, 2018; Ward & Webster, 2016). Consequently, grouping behavior influences individual and population level processes including fitness, genetic structure, and the transmission of information and disease (Archie et al., 2008; Rushmore et al., 2013; Silk, 2007).

In practice, groups represent the sampling unit of interest in many studies, such as those that investigate the underlying drivers of social behavior (Krause & Ruxton, 2002). Consequently, groups are often central to the study of animal behavior and behavioral ecology and, thus, their investigation necessitates a clear consensus on what represents a group. Yet, the concept of group suffers from inconsistent definitions and confusing use of terms which hinder effective scientific communication, evaluation of foundational ideas, and comparisons between studies (Jax, 2006; Nakazawa, 2020; Viscido & Shrestha, 2015).

Theoretical definitions provide broad interpretations of what constitutes a group. For example, Whitehead (2008) defined groups as “sets of animals that actively achieve or maintain spatiotemporal proximity over any time scale and within which most interactions occur” and Wilson (1975) defined them as “any set of organisms, belonging to the same species, that remain together for a period of time while interacting with one another to a distinctly greater degree than with other conspecific organisms.”. While such definitions are generally congruent and effectively encapsulate theoretical concepts of group, they are qualitative and have limited utility in the field as they provide no objective and reproducible means of assigning observed animals into groups (Viscido & Shrestha, 2015).

Consequently, researchers have developed operational definitions that are based on diverse empirical criteria, including spatial proximity, behavior, and directionality, with the resulting sets of individuals referred to by a surfeit of terms (e.g., *group*, *party*, *flock*, and *school*) (Gibson & Mann, 2009; Kasozi & Montgomery, 2020; Whitehead, 2008). For example, a 50 m threshold distance has been used to define *groups* of roe deer (*Capreolus capreolus*) (Pays et al., 2007), a 500 m radius from an estimated centre has been used to define *aggregations* of African savanna elephants (*Loxodonta africana*) (Wittemyer et al., 2005), while *shoals* of guppies (*Poecilia reticulata*) have been defined as individuals within four body lengths of one another (Croft et al., 2006). It should be noted that, although such definitions can effectively delimit spatial clusters of individuals, they do not necessarily identify the underlying driver (i.e., attraction between members of a group versus an external factor that gives rise to an aggregation) (Croft et al., 2008; Krause & Ruxton, 2002). Furthermore, a single group defined in such a way does not attest the presence of long‐term social bonds between group members (Gowans et al., 2007), instead, it represents an instantaneous observation of animals that is typically treated as a sampling unit (Table 1) (Connor et al., 1998; Farine & Whitehead, 2015; Wells et al., 1987).

**TABLE 1:** Broad definitions of groups that represent sampling units and social units and common criteria and analyses used to demarcate them.

	Sampling unit	Social unit
Broad definition	An instantaneous observation in the field of a set of individuals that maintain spatial proximity over a period of minutes to hours^a^	A set of individuals that display strong associations over a period of months to years^a^
Criteria to determine membership	Spatial proximity, behavior, direction of movement, level of coordination, and interaction^b^	Social network analysis, association indices (e.g., half weight index)^c^

^a^ The definitions are based on the definitions of *school* and *group* from Wells et al. (1987) and Connor et al. (1998); however, we chose to not use these terms to avoid confusion caused by inconsistency in how they are used in the literature.

^b^ Connor et al. (2000) and Gibson and Mann (2009).

^c^ Whitehead (2008).

Confusingly, several of the terms used to refer to such sampling units, including *group*, are also used to refer to social units—a related, but distinct, concept (Table 1). Social units vary considerably between species and populations, however, in general, they are sets of individuals that display strong and stable associations over periods of days to years (Connor et al., 1998; Wells et al., 1987; Whitehead, 2008). The distinction between these sampling and social units has been emphasized by some authors (e.g., Connor et al., 1998; Wells et al., 1987; Whitehead, 2008) and is evident in studies that use different terms to refer to each, for example, *groups*—*pods* of killer whales (*Orcinus orca*) (Baird & Dill, 1996; Esteban et al., 2016), *parties*—*communities* of chimpanzees (*Pan troglodytes*) (Rushmore et al., 2013), or *parties*—*core social groups* of African savanna elephants (Archie et al., 2006). Sampling and social units are often connected via the “gambit of the group” assumption, where membership in sampling units is used to assess the stability of individual associations in space and time, from which social units are then derived (Whitehead, 2008; Whitehead & Dufault, 1999). Importantly, social units can also be derived independently of such sampling units by analyzing, for example, nearest neighbors, time lags of photographic identifications, or behavioral interactions (e.g., grooming) (Croft et al., 2008; Tavares et al., 2022; Whitehead, 2008). In this study, for clarity, we follow the broad definitions outlined in Table 1 to distinguish groups that represent sampling units and social units. Moreover, unless otherwise stated, we use the term *group* to refer to a sampling unit of individuals observed in the field (Table 1).

**FIGURE 1:**
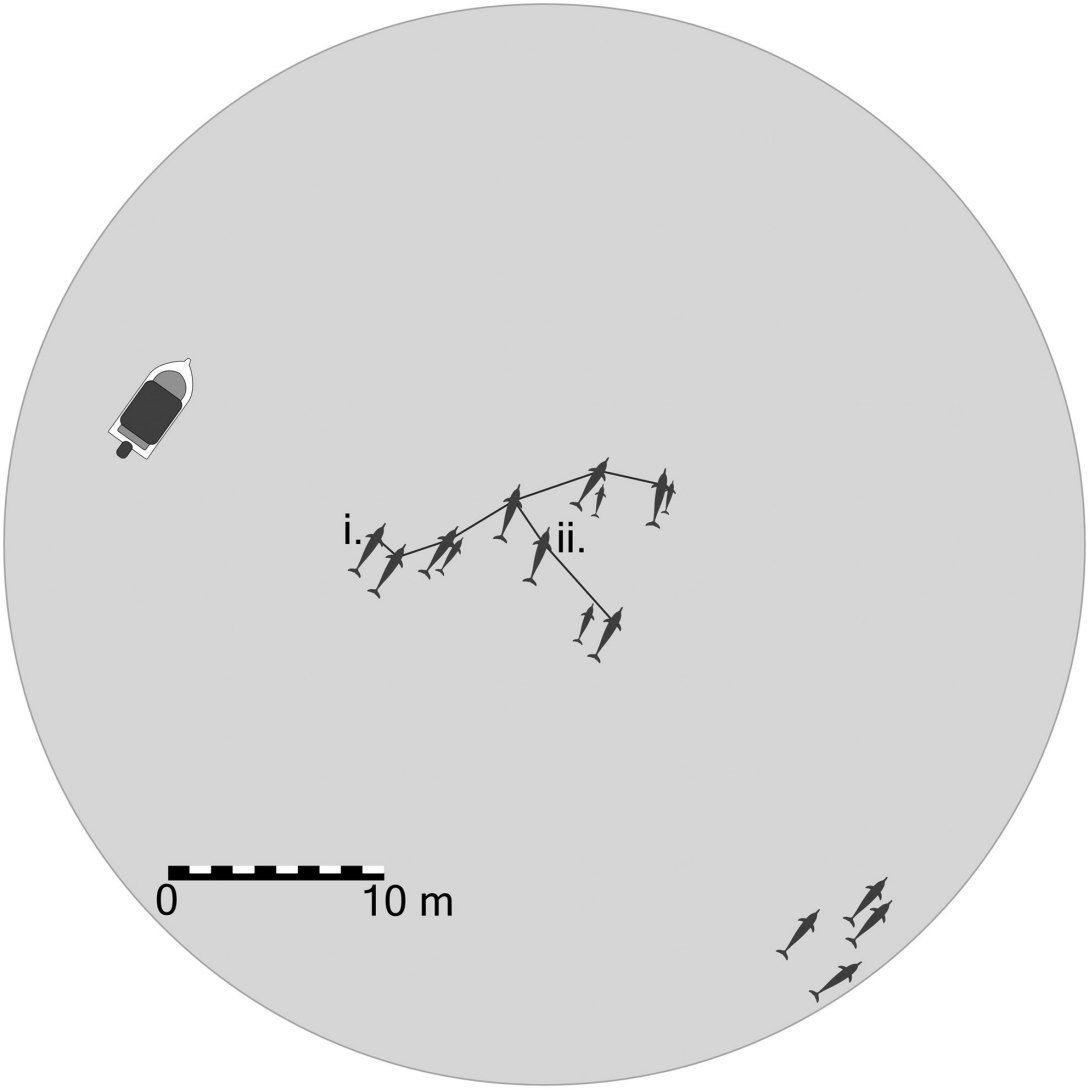
Two ways that spatial proximity rules are applied in group definitions: a 10 m chain rule, represented by the lines beginning from the individual closest to the research vessel (i) and extending to all those within 10 m of another group member; and a 25 m focal individual rule, represented by the shaded circle, where all individuals within 25 m of a focal individual (ii) are considered members of the same group. Note that the two definitions result in distinct delimitations of group with different data on measures of sociality. For example, the 10 m chain rule results in a group of 12 individuals, while the 25 m focal individual rule results in a group of 16.

Groups often form the basis for the collection and analysis of data and, thus, they are a fundamental part of the study methods and should be explicitly and appropriately defined (Franks et al., 2010; Gibson & Mann, 2009; Mann, 1999; Martin & Bateson, 2007). Nevertheless, the issue of how exactly to operationally define groups has proven problematic (Aureli et al., 2012; Kasozi & Montgomery, 2020; Viscido & Shrestha, 2015). This is, in part, due to the contrasting requirements of two opposing, yet valid, arguments. On the one hand, it has been argued that group definitions should be standardized, thus enhancing our potential to draw broad conclusions from comparative studies (Dudzinski et al., 1993; Kasozi & Montgomery, 2020; Viscido & Shrestha, 2015). Disparate definitions may produce contrasting results whose differences are attributable to methodological inconsistencies, rather than variation in behavior (Connor et al., 2000; Viscido & Shrestha, 2015). For example, in the situation illustrated in Figure 1, the 10 m chain rule and the 25 m focal individual rule result in two different delimitations of group with divergent data on measures of sociality (e.g., group size, number of associates, and sex/age composition). Thus, the definition of a *group* influences the nature of the data obtained, potentially reducing comparability between studies whose definitions are incompatible (Connor et al., 2000; Gibson & Mann, 2009; Gygax, 2002; Viscido & Shrestha, 2015). On the other, it has been argued that group definitions should be tailored to study populations and questions in order to account for differences in the biology of the population (e.g., grouping dynamics, size, and communication range) and the study methods (Kasozi & Montgomery, 2020; Krause & Ruxton, 2002). For studies of similar species, a reasonable compromise between these two arguments should be possible, allowing for increased comparability between studies without detracting from the relevance of the definitions (Dudzinski et al., 1993; Kasozi & Montgomery, 2020; Viscido & Shrestha, 2015).

In addition to balancing the aforementioned arguments, group definitions should be biologically meaningful to the animals to which they are applied (Croft et al., 2008; Krause & Ruxton, 2002). Approaching study questions from the perspective of the individuals involved can provide insight into grouping behavior and resulting social organization (Aureli & Schino, 2019). To achieve this, definitions should, ideally, be derived from empirical analyses of relevant parameters, such as interindividual distances, coordination, or communication range, which may vary between taxa (Croft et al., 2008; Krause & Ruxton, 2002; Whitehead, 2008). Finally, as groups represent sampling units, they should be defined in such a way that they are practical, objective, and reproducible in field research settings. By appropriately defining group, researchers improve their ability to collect meaningful and standardized data and, thus, strengthen the conclusions of their studies.

Delphinids are highly social animals and grouping behavior is an integral part of their life history, behavior, and ecology (Connor, 2000; Gowans et al., 2007; Gygax, 2002). Nevertheless, in published studies, group definitions are often absent, subjective, or arbitrary—a problem that is compounded by overlapping use of terms (Connor, 2000; Connor et al., 1998; Dudzinski et al., 1993; Gibson & Mann, 2009; Gygax, 2002). The issues surrounding delphinid group definitions have been raised by various authors: Connor et al. (2000) briefly reviewed group definitions applied to bottlenose dolphins (*Tursiops* sp.) and the ramifications of their differences on measures of group size; Mann (1999) emphasized the importance of defining group as part of sampling protocols; Syme et al. (2021) highlighted the need for explicit group definitions when studying mixed‐species groups; and Gibson and Mann (2009) discussed the different criteria that have been used to define groups in research on dolphins and primates, emphasizing that differences in group definitions hinder comparisons between studies. Nevertheless, there is still no consensus on which terms and criteria to use when defining delphinid groups and many definitions lack biological justification and are inadequate in certain situations. Moreover, possible solutions to these issues, such as the use of common terms and criteria or a standardized process to formulate definitions, are lacking.

We conducted a literature review of published studies and an online survey of the delphinid research community to: (1) determine which terms (i.e., *group*, *school*, *party*, and *pod*) are most commonly used, compare how they are defined, and propose recommendations for their future use; (2) assess the criteria in group definitions in terms of their use, relevance, and applicability; (3) identify issues associated with current group definitions as well as their potential solutions; and (4) propose a process for formulating group definitions. Due to the diversity of grouping behaviors that delphinids exhibit, a single definition that encapsulates all delphinid groups is unreasonable and unachievable. Accordingly, we do not aim for such a definition, but rather for the use of explicit group definitions that are appropriate for the study species and questions at hand, yet based on a set of common objective criteria and, preferably, derived via a standardized process. Such improved practices in defining delphinid groups will facilitate future socioecological research on these group‐living species.

## 2 METHODS

### 2.1 Literature review

We searched the citation database Scopus for studies on delphinids by using a search query composed of three parts: the 17 genera of the family Delphinidae (Society for Marine Mammalogy Committee on Taxonomy, 2020); the four key terms (i.e., *group*, *school*, *party*, and *pod*); and terms describing core research areas including *association*, *behavioral ecology*, *social network*, and *social organization* (see Appendix Item A1 for the complete search query). Additionally, the results were restricted to peer‐reviewed journal articles and book chapters published in English. The citation information of all results, including the abstracts, was downloaded using the Python package pybliometrics (Python Software Foundation, 2016; Rose & Kitchin, 2019).

We filtered the results by reading the abstracts to keep only those studies that either included visual observations of delphinid groups, used delphinid groups or a derived value (e.g., group size or membership) as a factor in analysis, or reviewed and discussed delphinid social groups. Studies that were exclusively based on data obtained remotely (e.g., passive acoustic monitoring) or from dead animals (e.g., necropsies) were removed, as were studies of captive delphinids because they do not represent natural grouping behavior. By this process, we limited the review to studies where a definition of one or more of the key terms was likely to be necessary and present.

The full texts of the retained results were then downloaded and automatically searched for definitions of the key terms using custom‐written scripts in Python (Python Software Foundation, 2016). Where the automatic search encountered no definitions, the text was manually searched to verify if any definitions were present and had been overlooked. All definitions were then extracted and compiled along with information concerning the publication, including the year of publication and the study species. Finally, any studies that were cited in the extracted definitions but missed by the initial search were downloaded, filtered, and examined for definitions by the same procedure as described above. This tracing process was repeated as many times as necessary to ensure that citations were traced back to the original study.

### 2.2 Online survey

To further understand how delphinid researchers define the key terms and to provide an opportunity for direct comment, we conducted an online survey using Qualtrics Software (Qualtrics, 2020). The respondents were asked whether they were familiar with each term and, if they were, whether they considered it a synonym of any of the others. The respondents then defined the term, listed any scientific publications as a reference for their definition, and provided details of any difficulties that they had experienced with applying the definition. Additionally, the respondents were asked to state their main study species (see Appendix Item A2 for the full survey). The survey was circulated via the MARMAM mailing list (https://lists.uvic.ca/mailman/listinfo/marmam), an email list for marine mammal researchers (about 15,000 members in January 2020). Additionally, the same survey was sent to attendees of a workshop held at the 2019 World Marine Mammal Science Conference entitled *Sociality in riverine*, *lagoon‐living*, *and coastal cetaceans: A descriptive analysis*, where this topic was discussed. The survey was open for voluntary completion during a period of 3 months, at which point all responses were recorded. Responses that were incomplete or that did not contain at least one explicit definition were removed from further analysis. The online survey was conducted under approval from the Human Research Ethics Committee of Flinders University, South Australia.

### 2.3 Analysis

In order to gain an understanding of the use and acceptance of the key terms, we recorded the number of times that they were defined in the reviewed studies and in the survey responses as well as how familiar they were to the survey respondents. Additionally, to quantify the overlap in the use of the key terms, we calculated the percentage of survey respondents that considered each term to be a synonym of each of the others. Where a survey respondent considered a term to be synonymous with a previously defined term, we transcribed the definition of the previously defined term to the synonym.

All the definitions from the literature review and the online survey were classified as either sampling or social units based on the broad definitions and criteria listed in Table 1. In other words, definitions that were based on criteria such as spatial proximity and behavior and that were applied to individuals observed in the field were classified as sampling units, whereas definitions based on analyses conducted post‐sampling to establish patterns of association between individuals were classified as social units. This was necessary because sampling and social units are distinct concepts and, consequently, they are not comparable. Making this distinction also served to compare how the key terms are employed.

As they are not the focus of this review, definitions that were classified as social units were excluded from the following analysis of criteria. Due to low sample size (*n* ≤ 40) and the high rate of synonymy with *group*, the definitions of *school*, *party*, and *pod* as sampling units were also excluded from the following analysis of criteria.

The definitions of *group* as a sampling unit, however, were evaluated in terms of the criteria that they included to determine which criteria were considered most important. To achieve this, we followed an evaluation process (Table A1) to determine which of eight nonmutually exclusive criteria each definition contained (i.e., spatial proximity, behavior, movement and directionality, number of individuals, visual range of the observers, interactions, temporal proximity, and species present). This involved assessing whether the definitions contained any parts that met the requirements for each criterion. Finally, to understand the origins of the definitions, we recorded the scientific publications that were cited in support of each definition.

## 3 RESULTS

### 3.1 Literature review and online survey

**FIGURE A1:**
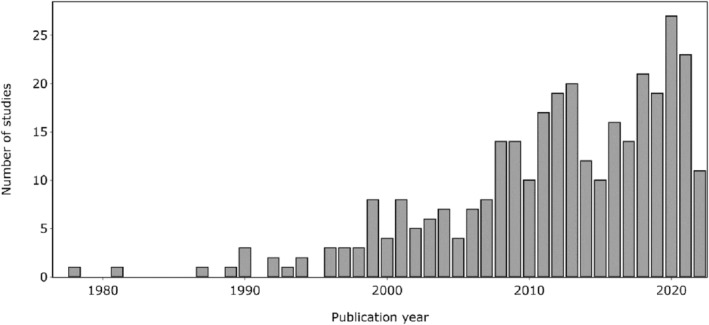
The publication year of the studies included in a review on the definition of delphinid groups.

**FIGURE A2:**
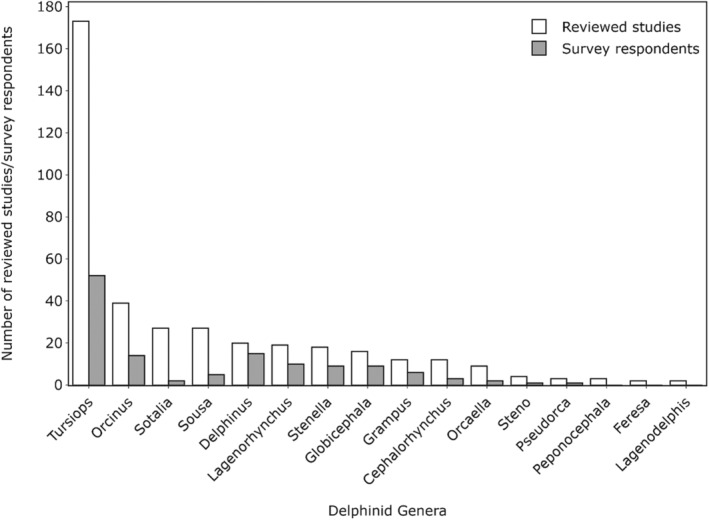
Number of reviewed studies and survey respondents that studied each of the listed delphinid genera.

The initial literature search returned 1662 studies to which a further 63 were added after reviewing the citations within the definitions. This amounted to a total of 1725 studies, nearly half of which (707 studies, 41.0%) were retained after the filtering process. Of the studies that were retained, 325 (46.0%) contained an explicit definition of one or more of the key terms and were kept for further analysis (hereafter, “reviewed studies”). These reviewed studies covered over four decades of research (1978–2022) (Figure A1) on a diverse range of delphinids, totalling 32 species from 16 genera (Figure A2). Most studies (173 studies, 52.2%), however, focussed on the genus *Tursiops*.

**FIGURE A3:**
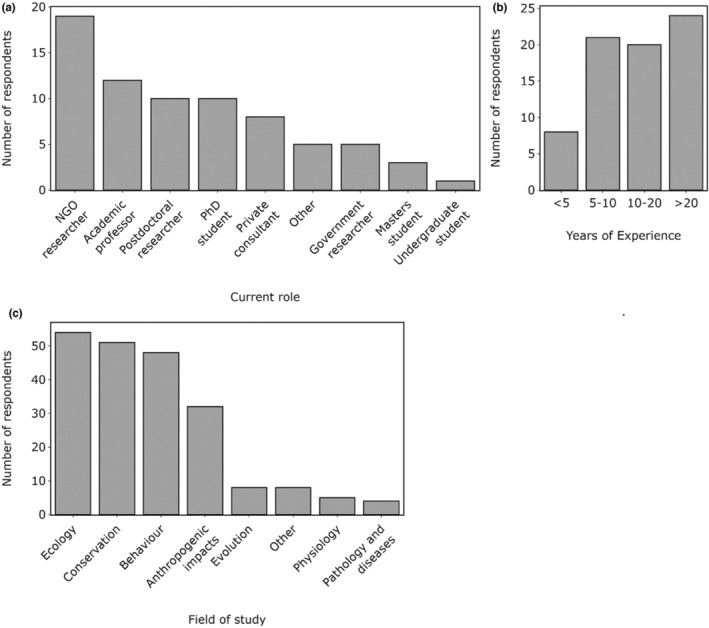
Details of the respondents to an online survey on the definition of delphinid groups showing (a) their current role, (b) their number of years of experience studying delphinids, and (c) their principal fields of study.

From the online survey, we received a total of 214 responses, 73 (34.1%) of which contained one or more explicit definitions of the key terms (hereafter, “survey responses”). The survey responses were primarily completed by NGO researchers (19 respondents, 26.0%), academics (12 respondents, 16.4%), post‐doctoral researchers (10 respondents, 13.7%), PhD students (10 respondents, 13.7%), and private consultants (8 respondents, 11.0%) with the remaining categories each accounting for <10% of respondents each (Figure A3a). The respondents mostly had more than 5 years of experience researching delphinids (65 respondents, 89.0%) (Figure A3b), chiefly in the fields of ecology, conservation, and behavior (Figure A3c). The principal study species of the survey respondents were also varied, covering 24 species from 13 genera, with the genus *Tursiops* being predominant (52 respondents, 71.2%) (Figure A2).

### 3.2 Familiarity and synonyms

**FIGURE A4:**
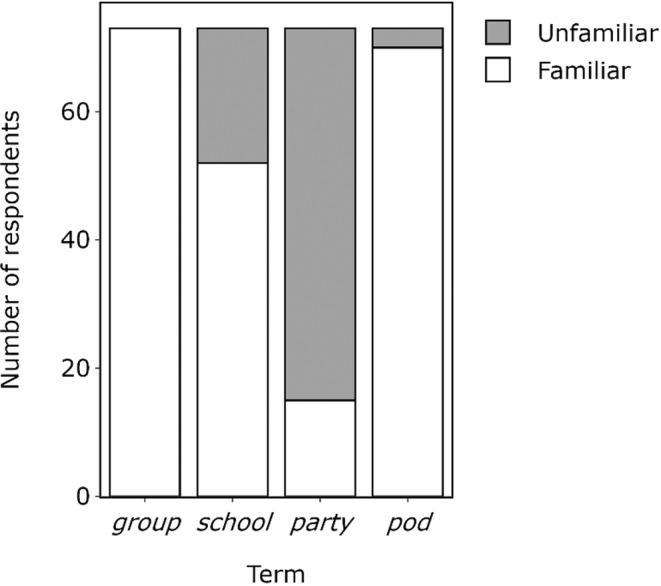
Number of respondents to an online survey of delphinid researchers who were familiar or unfamiliar with four key terms that are used to refer to delphinid groups.

The terms *group* and *pod* were the most familiar to the survey respondents, with 73 (100.0%) and 70 (95.9%) respondents familiar with them, respectively (Figure A4). The term *school* was familiar to 52 (71.2%) respondents, while *party* was only familiar to 15 (20.6%) (Figure A4). The term *party* was considered to be synonymous with *group* by most of the respondents who were familiar with this term (11 respondents, 73.3%), while *school* and *pod* were considered to be synonymous with *group* by approximately half of the respondents who were familiar with them (*school*: 27 respondents, 51.9%; *pod*: 35 respondents, 50.0%).

### 3.3 Definitions

The reviewed studies provided a total of 344 definitions, mostly of the term *group* (286 definitions, 83.1%), with relatively few definitions of *school* (29 definitions, 8.4%), *pod* (24 definitions, 7.0%), and, in particular, *party* (5 definitions, 1.5%). Similarly, the survey responses contained 192 explicit definitions, mainly of the term *group* (69 definitions, 35.9%), followed by *pod* (66 definitions, 34.4%), *school* (43 definitions, 22.4%), and *party* (14 definitions, 7.3%).

**FIGURE A5:**
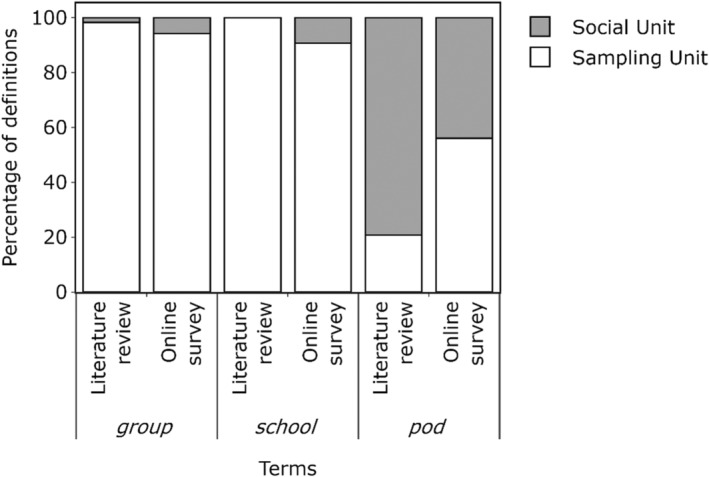
Percentage of definitions obtained from a review of the delphinid literature and an online survey of delphinid researchers that treated the terms *group*, *school*, and *pod* as sampling and social units.

In both the reviewed studies and the survey responses, the clear majority of definitions of *group* (reviewed studies: 281 definitions, 98.3%; survey responses: 65 definitions, 94.2%) and *school* (reviewed studies: 29 definitions, 100.0%; survey responses: 39 definitions, 90.7%) represented sampling units (Figure A5). In contrast, *pod* was principally defined as a social unit in the definitions from the reviewed studies (19 definitions, 79.2%) and as a sampling unit by just over half of the definitions from the survey responses (37 definitions, 56.1%) (Figure A5).

### 3.4 Criteria used to define group

**FIGURE 2:**
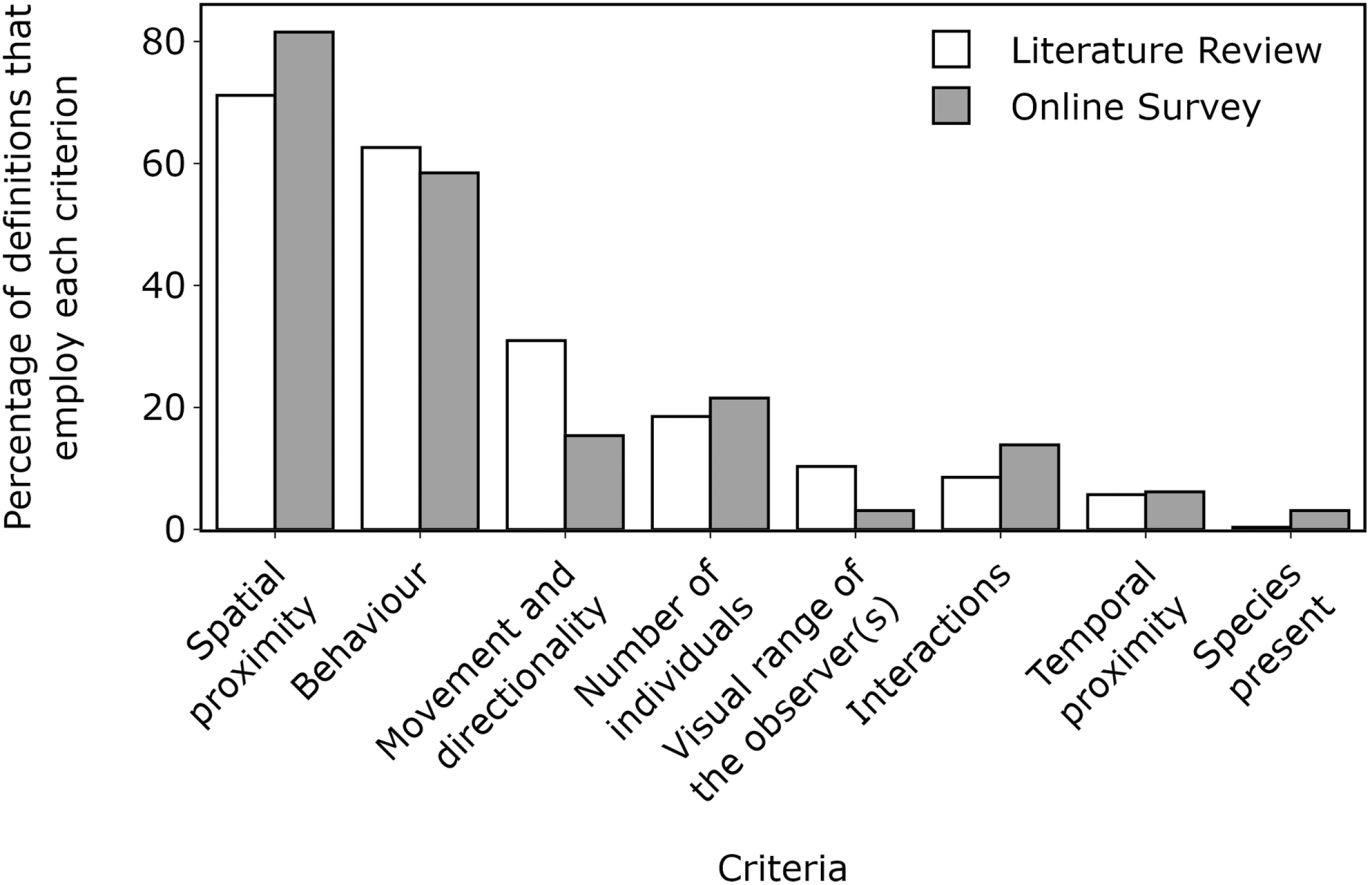
Percentage of definitions, obtained from a literature review and an online survey, that employed each of several criteria to determine membership in delphinid groups.

When defining *group* as a sampling unit, the spatial proximity of individuals was the most used criterion in the definitions from both data sets (reviewed studies: 200 definitions, 71.2%; survey responses: 53 definitions, 81.5%) (Figure 2). Spatial proximity was measured with 32 different rules (e.g., 10 m chain rule) in the reviewed studies and eight in the survey responses. Among these rules, distances between individuals were typically estimated using either a chain rule, a fixed‐point rule, or a focal individual rule (Figure 1), while the threshold distances that were employed varied largely, from 5 m to 10 km, and were estimated in both standard units (e.g., metres) and relative units (e.g., body lengths).

The behavior of the animals was the second most common criterion in definitions of *group* from both datasets (reviewed studies: 176 definitions, 62.6%; survey responses: 38 definitions, 58.5%) (Figure 2). Although the criteria were worded in numerous ways, they typically required the individuals to be engaged in the same or similar behavior in order to be considered members of the same group. Additionally, from the reviewed studies, 87 definitions (31.0%) considered that animals in a group must move in the same direction as did 10 (15.4%) from the survey responses (Figure 2).

From the reviewed studies, 52 definitions (18.5%) specified the number of individuals as a criterion for the definition of *group* as did 14 (21.6%) from the survey responses. These definitions were principally divided into those that stated that any number of individuals can constitute a group (reviewed studies: 36 definitions, 69.2%; survey responses: 2 definitions, 14.3%) and those that specified that a group must contain two or more individuals (reviewed studies: 16 definitions, 30.8%; survey responses: 9 definitions, 64.3%). The remaining criteria (i.e., the visual range of observers, interactions, temporal proximity, and species present) were employed in only a few definitions (<15%) from both data sets (Figure 2).

### 3.5 Studies cited to support the definition of group

Of the definitions of *group* from the reviewed studies, 206 (73.3%) contained citations of the peer‐reviewed literature. In total, 83 different publications were cited to support the group definitions. We considered four of these—Shane (1990), Smolker et al. (1992), Irvine et al. (1981), and Wells et al. (1987)—to be key references as they were cited in more than 5% of the definitions (Table 2). It is worth noting that these four references, and their group definitions, are based on studies of a single delphinid genus: *Tursiops*.

**TABLE 2:** The key studies cited in definitions of *group* (i.e., cited in >5% of the definitions from the reviewed studies) and their original definitions.

Original definition	Study	Number of citations in the definitions from the reviewed studies
*A* pod *was defined as any group of dolphins observed in apparent association, moving in the same direction and often, but not always, engaged in the same activity* ^a^	Shane (1990)	56 (19.9%)
*Operationally, a dolphin was considered to be a member of a* party *if it was within 10 meters of any other member (a 10 meter “chain” rule)* ^a^	Smolker et al. (1992)	55 (19.6%)
*Consequently, all bottlenose dolphins sighted within about 100 m of the boat were defined as a* group	Irvine et al. (1981)	21 (7.5%)
*Dolphins sighted within an area of approximately 100 m radius were considered to be in a single* school *(=*group, Irvine et al., 1981 *)* ^a^	Wells et al. (1987)	16 (5.7%)

^a^ Note that the original definitions, except that of Irvine et al. (1981), are not for the term *group*, but rather for the terms *pod*, *party*, and *school*.

## 4 DISCUSSION

When considering the diversity of grouping behaviors that are exhibited by the numerous delphinid species, it is evident that an all‐encompassing definition of group that can be applied to all species, situations, and studies is an unrealistic goal (Kasozi & Montgomery, 2020; Krause & Ruxton, 2002). Nevertheless, there is potential to consolidate terms and criteria and strengthen the biological basis of delphinid group definitions, thus reducing inconsistency and improving comparability between definitions while increasing their biological relevance and maintaining their utility in the field. Given the wide variety of studies and researchers that rely on groups of dolphins as their sampling units, we acknowledge that our sample of definitions may not be all‐inclusive. However, we believe that through our comprehensive literature review and online survey we have obtained a representative sample of how delphinid groups are defined by researchers, allowing us to assess the key terms and criteria to ultimately make recommendations for their future use. Furthermore, we highlight some of the challenges and considerations that researchers are faced with when defining delphinid groups and propose a standardized process, based on objective criteria, to facilitate the formulation of biologically meaningful definitions, thus assisting future studies.

### 4.1 The need for explicit definitions

The first issue encountered was the frequent lack of an explicit definition of the term used to refer to delphinid groups, with over half of the retained studies not providing one. Therefore, we reaffirm previous recommendations that, for each term used, authors explicitly state the definition of the term and any justification for the choice of term and definition (Dudzinski et al., 1993; Mann, 1999; Martin & Bateson, 2007).

### 4.2 Bias towards *Tursiops* in commonly used definitions

Among the definitions that we obtained, from both the review and the survey, there was a notable bias toward studies of the genus *Tursiops*. This is, perhaps, unsurprising given that both *Tursiops* species, particularly the common bottlenose dolphin (*Tursiops truncatus*), have widespread, typically coastal, distributions, and are often the focus of research (Jarić et al., 2015; Wang, 2018; Wells & Scott, 2018). Furthermore, the four most common citations in the *group* definitions—Shane (1990), Smolker et al. (1992), Irvine et al. (1981), and Wells et al. (1987)—were studies of coastal *Tursiops* populations. Nevertheless, these definitions were applied to other species. For example, the 10 m chain rule of Smolker et al. (1992) was applied to 14 species from 12 genera. This may be problematic if the grouping dynamics of *Tursiops* are not representative of the other species. To avoid the risk of projecting inferences taken from a certain species onto incompatible species, research on group formation needs to be more inclusive, particularly given the diversity in delphinid grouping behavior.

### 4.3 Terms used to refer to delphinid groups

A principal source of confusion when discussing delphinid groups is the inconsistent use of terms. The most familiar and commonly defined term in studies of delphinids is *group*, which has widespread use as a sampling unit. The term *school* has been used alongside *group* as a means of differentiating between sampling and social units (Connor et al., 1998; Wells et al., 1987), however this distinction appears to be somewhat blurred. Additionally, it appears that *school* has fallen into disuse amongst delphinid researchers, potentially due to its connotations of and connections to fisheries studies. The term *party* is not commonly used in studies of delphinids, despite its use in studies of primates and elephants (e.g., Archie et al., 2006; Machanda et al., 2013; Rushmore et al., 2013). Finally, unlike the other terms, *pod* is employed chiefly as a social unit, particularly when referring to stable, long‐lasting units of genetically‐related individuals, such as pods of killer whales.

These trends lead us to make two recommendations regarding the future use of the terms *group*, *school*, *party*, and *pod* in studies of delphinids. First, we recommend that *group* be used exclusively to refer to sampling units because it was the most familiar and most commonly defined term and was considered as a sampling unit by the majority of definitions. Second, we recommend that *pod* be applied solely to stable social units of genetically related individuals and that other types of social unit be defined by different terms (i.e., not *group* nor *pod*), for example, *band*, *clan*, or *alliance*, depending on the species' social organization. We believe that this is justified because, although it was not unanimous in the survey responses, there is clearly a strong tendency to use *pod* to describe such social units. Moreover, limiting the terms *group* and *pod* to one use and ceasing to use less frequent terms (i.e., *school* and *party*) will increase consistency and clarity.

### 4.4 Delphinid group definitions

When discussing delphinid group definitions, we focus specifically on the operational definitions that are applied in the field to delimit groups of delphinids that are typically treated as sampling units (Table 1). We follow previous work in advocating for definitions that: reconcile the contrasting requirements of the need to standardize definitions and the need to tailor definitions to individual studies; are derived from a relevant biological parameter and are, thus, biologically meaningful to the animals being studied; and are practical, objective, and reproducible, thus allowing for accurate sampling in the field (Aureli et al., 2012; Croft et al., 2008; Kasozi & Montgomery, 2020; Viscido & Shrestha, 2015). Such definitions increase the capacity of field biologists to make meaningful observations.

### 4.5 Criteria used to define delphinid groups

Although no single delphinid group definition can be applied universally, it would be feasible for studies of delphinids to, at least, base their definitions on shared criteria. This would ensure a degree of compatibility between them while allowing for some necessary variation to account for differences between study populations. Accordingly, to determine which criteria are most appropriate, we evaluate the four most commonly used criteria in definitions of delphinid groups (i.e., spatial proximity, behavior, movement and directionality, and the number of individuals) with regard to their use among delphinid researchers and, more broadly, in studies of animal behavior and ecology; their relevance to the biological concept of group; and their applicability in the field.

**FIGURE 3:**
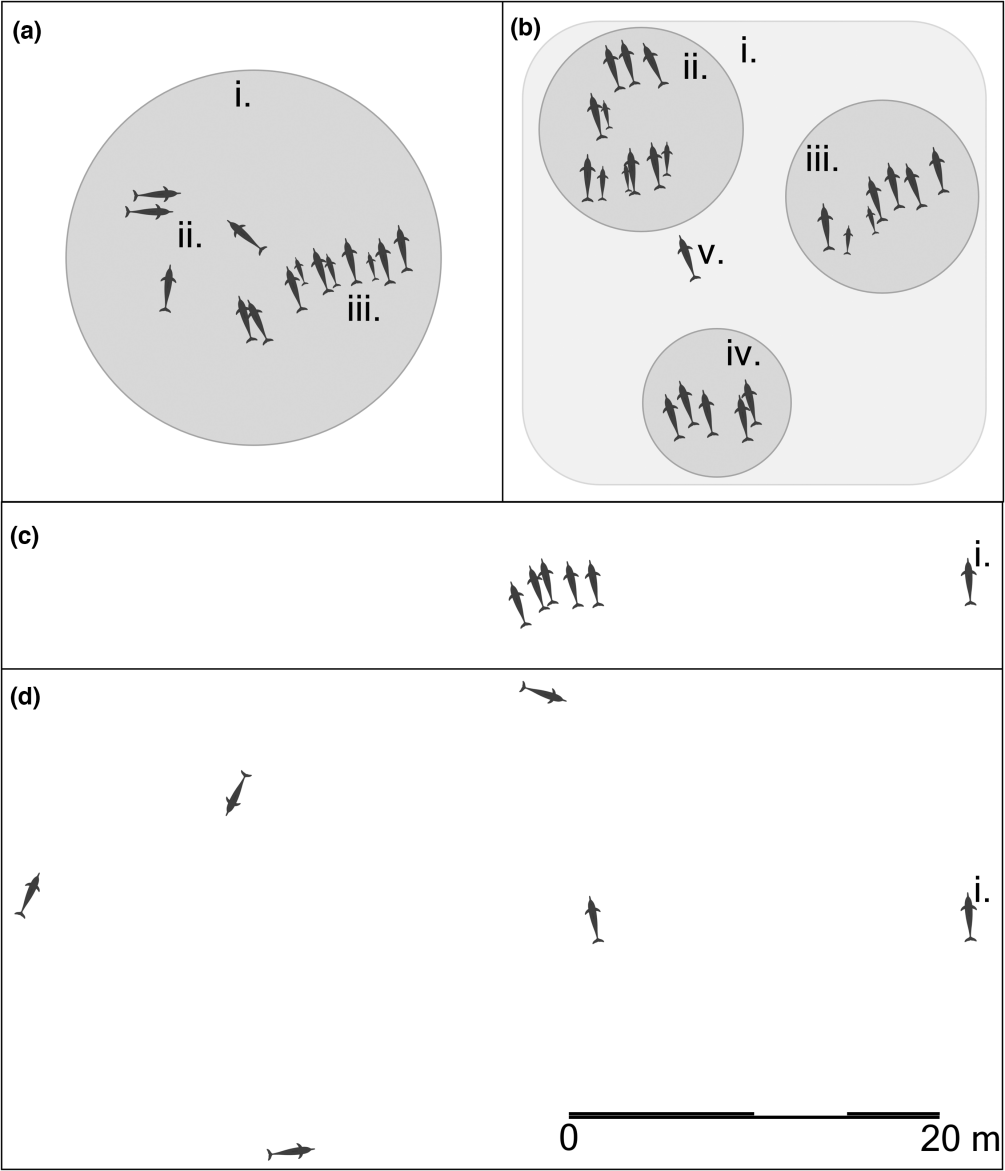
Various situations that challenge delphinid group definitions: (a) animals form a distinct spatial cluster (i) but differ in their behavior (e.g., socializing, ii, and resting, iii); (b) several subgroups (ii, iii, and iv) form a single large group (i) and individuals move between subgroups (v); (c,d) changes in cohesiveness affect the relative importance of fixed threshold distances such that the sixth individual (i) is an outlier when the other five are resting (c), but not when they are foraging (d) despite being separated by the same distance (20 m) from its nearest neighbor in both situations.

Spatial proximity among individuals is regarded as an essential criterion for delimiting group membership (Croft et al., 2008; Kasozi & Montgomery, 2020; Krause & Ruxton, 2002; Whitehead, 2008) and was included in over two‐thirds of the definitions of *group* from both the reviewed studies and the survey responses. Furthermore, spatial proximity can be quantified with rules that can be readily and objectively reproduced in the field (e.g., 10 m chain rule, 100 m chain rule). These rules are similar in principle and construct to those found in studies of other taxa (e.g., Archie et al., 2006; Castles et al., 2014; Kasozi & Montgomery, 2020; Machanda et al., 2013; Rushmore et al., 2013; Wittemyer et al., 2005). Nevertheless, we encountered several issues regarding the use of spatial proximity to define delphinid groups: first, there are certain situations where fixed measures of spatial proximity are difficult to apply and may not adequately capture the complexity of spatial organization (Figure 3); second, some threshold distances do not consider the practical limitations inherent to visual observations of delphinids; and, third, we found little biological justification for the choice of spatial proximity rules. Nevertheless, we believe that these issues can be overcome by careful formulation of a definition and we dedicate the following sections of this article to discussing them and their potential solutions in more detail.

Behavior was commonly used in definitions of *group*, while the direction and movement of the observed animals was used as a criterion in only a few definitions, almost always alongside a behavior criterion and often in accordance with the definition given by Shane (1990) (Table 2). Behavior and directionality are not usually mentioned in theoretical group definitions (e.g., Ward & Webster, 2016; Whitehead, 2008; Wilson, 1975) and are only occasionally employed in group definitions of other taxa (Kasozi & Montgomery, 2020). Additionally, the vagueness and subjectivity of some behavior criteria (e.g., “often, but not always, engaged in the same activity”) allow for inconsistencies to arise (Kasozi & Montgomery, 2020; Mann, 1999). The directionality criteria were similarly subjective and no means of quantifying directionality was provided. Furthermore, survey respondents reported difficulties in applying behavior and directionality criteria when animals in close proximity form a distinct, cohesive spatial unit but differ in their behavioral state and direction of movement (Figure 3a) and when animals are spread out so far that their behavior and movements cannot be reliably noted. Given the subjectivity and the lack of quantification of directionality criteria, we recommend excluding them from group definitions. Behavior criteria also entail certain complications, however, given the influence of behavior on interindividual distances and, thus, spatial proximity, we recommend that behavior be incorporated into the formulation of group definitions (Aureli et al., 2012; Denardo et al., 2001).

The final issue concerns the number of individuals that constitutes a group (Dudzinski et al., 1993; Krause & Ruxton, 2002). A few definitions specified that a group could contain any number of individuals (i.e., including single individuals), while a few stated that a group must contain two or more individuals. Most definitions, however, did not explicitly state a number of individuals required to constitute a group. Common usage of the word *group* implies the presence of multiple individuals and, from a biological perspective, a single individual cannot display the fundamental features of a group (e.g., proximity, attraction, and interaction among individuals). Therefore, we do not consider that a single individual constitutes a group. Nevertheless, single individuals are important when considering ecological questions, and they should be included in relevant analyses so as to not bias the results (Dudzinski et al., 1993, Krause & Ruxton, 2002). A potential solution, as mentioned in the survey responses and employed in some of the reviewed studies (e.g., Karczmarski, 1999), is to use multiple terms, for example, *singleton* (single individual), *group* (two or more individuals), and *sighting* (singletons and groups), the latter of which can then be used in analysis.

### 4.6 Challenges faced when defining groups and their potential solutions

Although spatial proximity is key when defining delphinid groups, we identified several issues concerning its use. First, certain situations make it difficult to apply spatial proximity rules. Numerous delphinids are known to, at times, display multiple levels of spatial organization (Figure 3b). For example, spinner (*Stenella longirostris*) and pantropical spotted dolphins (*Stenella attenuata*) form supergroups containing multiple groups (or subgroups) that each maintain their respective boundaries (Kiszka et al., 2011). In these situations, group, which represents a single level of spatial organization, will not necessarily capture the full complexity of the animals' grouping dynamics. Consequently, it may be necessary to define multiple levels of grouping with unique terms (e.g., *subgroup*, *group*, and *supergroup*).

Furthermore, the cohesiveness of delphinid groups varies, often according to behavior. For example, animals may form tight groups to rest and spread out to forage (Gowans et al., 2007). Changes in group spacing can render a single, fixed threshold distance inadequate because the importance of that distance changes according to the spacing among individuals (Miller & Gerlai, 2011). If, for example, five individuals are resting with an average nearest neighbor distance of 1 m, then, intuitively, a sixth individual 20 m from them is not part of their group (Figure 3c). If, on the other hand, five individuals are foraging with an average nearest neighbor distance of 25 m, then, intuitively, a sixth individual 20 m from the closest of them is part of their group (Figure 3d). Assuming that our intuition regarding group membership is accurate, a 10 m chain rule would correctly separate the sixth individual in the first situation, but would incorrectly separate it, and many others, in the second. Conversely, a 50 m chain rule would incorrectly include the sixth individual in the first situation but would correctly include all individuals in the second. What is required, then, is a flexible spatial proximity rule that can account for changes in cohesiveness (e.g., one with a different distance threshold for each behavioral state) (Miller & Gerlai, 2011).

Although group definitions should be based on relevant biological parameters, we cannot be oblivious to the practical limitations that we, researchers, are subject to. For example, any visual observation of a delphinid group is inherently limited by the visual range of the observers. Particularly when larger threshold distances are employed (e.g., 1000 m), certain individuals that meet the spatial proximity requirements may be beyond the limits of reliable observation or even detection. Moreover, to mitigate potential biases introduced by visual assessment of distances over water, researchers should periodically train field observers by estimating distances over water to objects placed at known intervals, as determined with a line or rangefinder. Thus, the limitations of visual observations should be considered and minimized when developing and applying definitions of group.

Finally, and perhaps most importantly, when defining group, we must consider what is biologically meaningful to the animals (Croft et al., 2008; Kasozi & Montgomery, 2020; Viscido & Shrestha, 2015). Yet, of the 200 definitions from the reviewed studies that employed a spatial proximity criterion, only four justified their distance on a measured parameter (acoustic communication range: Parsons et al., 2009, Foster et al., 2012; and nearest neighbor distances: Visser et al., 2014, 2017). This raises the question: are the distances used to define groups appropriate for the species to which they are applied?

Two studies—Parsons et al. (2009) and Foster et al. (2012)—based their spatial proximity criterion on the estimated acoustic communication range of the study species, resulting in a distance threshold of 10 km. Such an approach is advantageous because it is based on a variable that is intrinsically linked to group formation. There are, however, certain drawbacks. Active space (i.e., the range at which an acoustic signal can be detected and recognized) is known to vary according to numerous environmental (e.g., substrate type, sea state, salinity, and water depth) and biological variables (e.g., frequency, species, and position of the animal in the water column) (Janik, 2000; Quintana‐Rizzo et al., 2006). Accordingly, the estimated active space of delphinid whistles ranges from several 100 m to 25 km (Janik, 2000; Miller, 2006; Quintana‐Rizzo et al., 2006). This poses two issues. First, a threshold distance based on an active space calculated under certain conditions may not be relevant under different conditions. Second, the individuals in a group that is delimited by acoustic communication range are likely to be dispersed over an area that is too large to be reliably observed visually.

It seems, then, that a threshold distance that permits reliable visual observation of all group members is likely to fall within the limits of delphinid acoustic communication range and the corresponding group is likely, therefore, to include only a subset of all potentially interacting individuals. This is not an issue per se as the theoretical concept of group does not require the group to contain all interacting animals, but rather those that interact most (Whitehead, 2008; Wilson, 1975). Equivalent situations are found amongst other taxa. African savanna elephants, for example, recognize contact calls of family members at distances of up to 2.5 km (McComb et al., 2003), well beyond the distances that are used for determining group membership (e.g., 100 m: Archie et al. (2006); 500 m: Wittemyer et al. (2005)). It is necessary, however, to determine a point that marks a meaningful change in the level of interaction.

This can be achieved through empirical analyses of interindividual distances (Krause & Ruxton, 2002; Martin & Bateson, 2007; Whitehead, 2008). Clutton‐Brock et al. (1982), for example, analyzed the spacing of red deer (*Cervus elaphus*) and found that interindividual distances were distributed bimodally with a discontinuity around 50 m. This distance was verified by behavioral analysis and subsequently used to define red deer *parties* (Clutton‐Brock et al., 1982). Similar techniques have been conducted on spider monkeys (*Ateles geoffroyi*) with group behavior incorporated into the analysis to determine how it affects group spacing (Aureli et al., 2012; Ramos‐Fernández, 2005).

Two of the reviewed studies—Visser et al. (2014) and Visser et al. (2017)—achieved a similar outcome by selecting a threshold distance in situ based on the estimated distance from a focal individual to its nearest neighbor (Visser et al., 2014). This method is beneficial because it is adaptable to changes in cohesiveness; however, it is necessary to track a focal individual which could present challenges.

### 4.7 A proposed process for formulating delphinid group definitions

We believe that an ideal approach to improve delphinid group definitions is via the use of a standardized process by which researchers can formulate definitions for their study populations. Similar ideas have been recommended previously (Krause & Ruxton, 2002; Martin & Bateson, 2007; Whitehead, 2008) and employed in studies of ungulates (Clutton‐Brock et al., 1982), primates (Ramos‐Fernández, 2005), and fishes (Miller & Gerlai, 2008), but not, to our knowledge, in studies of delphinids. After studying these previously used techniques, evaluating the identified issues, and considering the unique challenges associated with delphinid research, we propose such a process. It involves analyzing interindividual distances to determine naturally occurring patterns that indicate appropriate distances at which to delimit groups.

**FIGURE 4:**
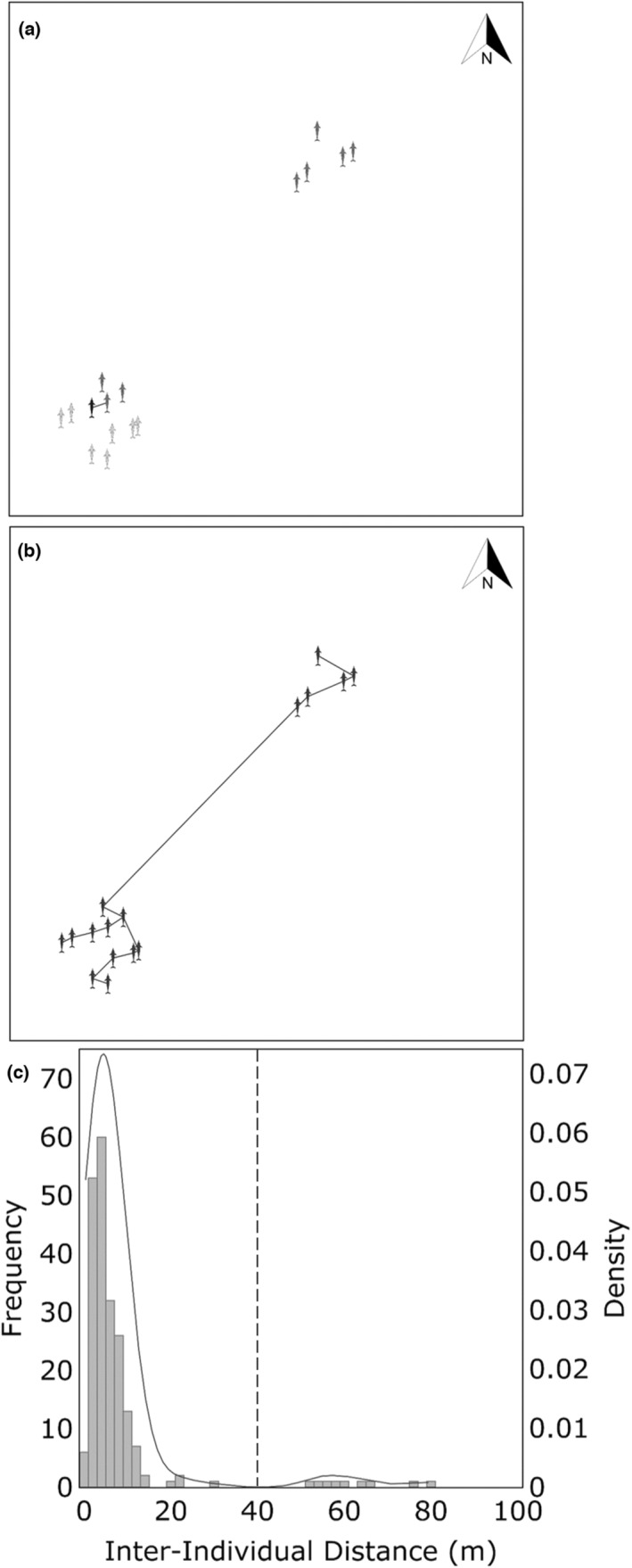
Simulated example of the proposed process for determining threshold distances for delphinid group definitions. The first step (a,b) is to measure within a given area the distances from each individual to its nearest neighbor in a constant direction (e.g., north). This involves (a) selecting a target individual (black), determining which other individuals are further north than the target individual (dark gray), then measuring the distance between the target individual and the nearest other individual that is further north (black line). This is then repeated for every individual within the observed area (b) so that all nearest northerly neighbor distances (black lines) are measured. Once the first step has been conducted on a sufficient number of samples, the frequency distribution of all the nearest northerly neighbor distances can be plotted (c) and a cutoff point (dashed line) can be determined by the presence of any discontinuities as determined by, for example, analyzing the gradient of a density curve.

Despite the difficulties associated with observing wild delphinids, photogrammetry using unmanned aerial vehicles (Dawson et al., 2017; Scott & Perryman, 1991) or portable stereo photogrammetry systems (Howland et al., 2012; Macfarlane et al., 2015) offers feasible ways of measuring interindividual distances. It is necessary to consider, however, which interindividual distances to measure. Possible options include the distances from each individual within a given area to its nearest neighbor in a constant direction (e.g., north) (Clutton‐Brock et al., 1982) (Figure 4a,b) or the distances from a focal individual to all others within a certain radius (Aureli et al., 2012; Ramos‐Fernández, 2005). The nearest neighbor in a constant direction option seems the most reasonable to us as nearest neighbors are arguably the most important in terms of interactions and maintenance of group cohesion (Ballerini et al., 2008; Miller & Gerlai, 2008; Partridge, 1981) while measuring in a constant direction captures any discontinuities in interindividual distances, rather than simply capturing the minimum distances between individuals (Figure 4). Moreover, nearest neighbor distances are typically what is tested by the commonly employed chain rules. The next step is to determine a threshold distance by plotting the distribution of the observed interindividual distances and finding a naturally occurring cutoff point (e.g., a discontinuity, Clutton‐Brock et al., 1982; or a steep decline, Ramos‐Fernández, 2005) by analyzing the gradient of a density curve (Figure 4c). Finally, it would be beneficial to incorporate behavior into any such investigation by considering its influence on spatial proximity (Aureli et al., 2012; Denardo et al., 2001). This can be achieved by either comparing the behavior of individuals allocated to the same or different groups (Clutton‐Brock et al., 1982) or by incorporating behavior as a factor in analysis (Aureli et al., 2012).

The result of this process would be a group definition based on a threshold distance that would be applied in the field as a chain rule. We recognize that the proposed process will not completely solve the issues associated with defining groups and that any definition produced via this process will still be susceptible to the problems that are inherent to observational delphinid research (e.g., observing animals that spend long periods underwater). Nevertheless, by utilizing techniques such as the proposed process, the issues associated with defining delphinid groups can be mitigated, thus improving the quality of the associated data. More specifically, group definitions produced via the proposed process would, first, be based on a biologically meaningful parameter that is relevant to the study population and would, second, be reproducible, objective, and practical, as has been recommended (Croft et al., 2008; Krause & Ruxton, 2002; Viscido & Shrestha, 2015; Whitehead, 2008). Furthermore, in situations where there are multiple levels of spatial organization, the distribution of interindividual distances would present multiple cutoff points, allowing for the determination of multiple threshold distances (e.g., one for subgroup and one for group). If behavior were incorporated as a factor, it would be possible to determine a threshold distance for each behavioral state. Thus, the resulting chain rule would be adaptable to the behavior and cohesiveness of the animals. If such a technique were standardized and applied to different species in diverse locations, then, although the resulting threshold distances would presumably vary, they would still be compatible because they would be formulated via the same process and would, therefore, represent the same aspect of grouping dynamics. Thus, we strike a compromise between the need to standardize definitions and the need to tailor definitions to the biology of each study population (Aureli et al., 2012; Kasozi & Montgomery, 2020; Krause & Ruxton, 2002; Martin & Bateson, 2007; Viscido & Shrestha, 2015).

We recognize that, due to the cost, training, and time required to implement this process, it will not always be feasible. If that is the case, we recommend that researchers use the term *sighting* as a sampling unit to refer to both *singletons* (i.e., single animals) and *groups* (i.e., two or more individuals within close spatial proximity). Moreover, *groups* should be defined by one of the two spatial proximity rules that were most commonly used in the definitions from both the reviewed studies and the survey responses: the 10 m chain rule or the 100 m chain rule (Table 2). Whether these distances are biologically meaningful is challenging to assess, yet, by using standardized terms and distances we can decrease inconsistency and improve comparability of published studies. Finally, we emphasize that, regardless of the term used, authors must provide an explicit formal definition.

## 5 CONCLUSIONS

Our literature review and online survey raise several issues concerning delphinid group definitions. Inconsistent use of terms and criteria hamper comparisons across species and locations. We believe that these difficulties can be overcome by a consensus on the use of terms and criteria. By analyzing definitions from a range of publications and by providing the delphinid research community with the opportunity to contribute via an online survey, we believe that we have pursued a democratic path toward such a consensus. Accordingly, the conclusions and recommendations that we provide are drawn from the perspectives of those who wrote the reviewed studies and completed the online survey. These perspectives, combined with broader work on animal grouping behavior, illustrate how we can address those issues that require further dedicated work to be minimized, such as the lack of justification and the inadequacy of group definitions in certain situations. We believe that our proposed process takes a step in the right direction by providing an empirical way of formulating biologically meaningful definitions that are compatible but that still account for variation in grouping dynamics. We hope that this review provides guidance to researchers and students in our field when confronted with the task of defining delphinid groups.

## AUTHOR CONTRIBUTIONS

**Jonathan Syme:** Conceptualization (equal); formal analysis (lead); investigation (lead); methodology (lead); writing – original draft (lead); writing – review and editing (lead). **Jeremy J. Kiszka:** Conceptualization (equal); formal analysis (supporting); investigation (supporting); methodology (supporting); supervision (supporting); writing – original draft (supporting); writing – review and editing (supporting). **Guido J. Parra:** Conceptualization (equal); formal analysis (supporting); investigation (supporting); methodology (supporting); supervision (lead); writing – original draft (supporting); writing – review and editing (supporting).

## CONFLICT OF INTEREST

The authors certify that they have no relevant conflict of interest.

## Supporting information


Supporting information S1

## Data Availability

The data from the literature review are available in the Supporting Information. The data from the online survey cannot be made publicly available as per the requirements of the Human Ethics approval.

## APPENDIX A

### Item A1 Search query used for a literature review on the definitions of delphinid *groups*, *schools*, *parties*, and *pods*

**Table:**

Delphinid genera		Key terms	Subject area keywords
*Orcaella*	*Delphinus*	group	abundance
*Orcinus*	*Lagenodelphis*	school	assoc*
*Globicephala*	*Sousa*	party	behav*
*Pseudorca*	*Lissodelphis*	pod	conservation
*Feresa*	*Lagenorhynchus*		distribution
*Peponocephala*	*Cephalorhynchus*		ecology
*Grampus*			network
*Sotalia*			site fidelity
*Steno*			soci*
*Tursiops*			space use
*Stenella*			residenc*

The query is composed of three parts: the 17 delphinid genera, the four key terms, and subject area keywords. The asterisk is a wildcard character, thus *soci** finds results for *social*, *sociality*, *social network*, etc.

The three parts (i.e., the *genera*, the *key terms*, and the *subject area keywords*) were joined in the following way:

TITLE‐ABS‐KEY (*genera*) AND TITLE‐ABS‐KEY (*terms*) AND TITLE‐ABS‐KEY (*study areas*).

This string was combined with limitations on language and document type to give the full query:

TITLE‐ABS‐KEY (orcaella OR orcinus OR globicephala OR pseudorca OR feresa OR peponocephala OR grampus OR sotalia OR steno OR tursiops OR stenella OR delphinus OR lagenodelphis OR sousa OR lissodelphis OR lagenorhynchus OR cephalorhynchus).

AND TITLE‐ABS‐KEY (group OR school OR party OR pod).

AND TITLE‐ABS‐KEY (abundance OR assoc* OR behav* OR conservation OR distribution OR ecology OR network OR “site fidelity” OR soci* OR “space use” OR residenc*).

AND LANGUAGE (“English”) AND DOCTYPE (“ar”) OR DOCTYPE (“ch”).

### Item A2 Online survey on the definition of delphinid *groups*, *schools*, *parties*, and *pods*

Information and Consent

In science, clear terminology provides a common basis of understanding, a solid foundation to develop concepts and theory, and facilitates scientific communication. In the literature on delphinid sociality, various definitions of group, school, party, and pod have been used to designate various concepts, often with inconsistent and unclear definitions. Variability among researchers and improper use of distinct terms generates confusion and hinders the development of comparative studies. Here we aim to investigate the definitions of these terms within our scientific community to assess which key criteria are more often used to define these terms and work toward clearer terminology and suggestions for defining the term group and associated terms.

We would appreciate it if you would kindly take 10–15 min to complete the following survey regarding your opinions on this subject. Your responses will remain anonymous. Thank you very much for your participation.

Please see the Participant Information Sheet if you require more information.

Do you give consent to take part in this project?

- Yes, I consent
- No, I do not consent

Personal information

What is your current role/position?

- Academic professor
- Postdoctoral researcher
- PhD student
- Masters student
- Undergraduate student
- Government researcher
- NGO researcher
- Private consultant
- other

What are your principal fields of study? (You may select more than one response)

- Ecology
- Physiology
- Behavior
- Evolution
- Conservation
- Pathology and diseases
- Anthropogenic impacts
- other

How many years of experience do you have studying delphinids?

- less than 5 years
- 6–10 years
- 11–20 years
- more than 20 years

Which delphinid species have you mainly studied?

Definition

With regards to the study of wild delphinids, are you familiar with the term group?

- Yes
- No

Please define group

Please list any scientific paper(s) that you use as a basis for your definition of group.

What difficulties have you experienced in applying this definition?

With regards to the study of wild delphinids, are you familiar with the term school?

- Yes
- No

Do you consider school to be a synonym of group?

- Yes
- No

Please define school

Please list any scientific paper(s) that you use as a basis for your definition of school.

What difficulties have you experienced in applying this definition?

With regards to the study of wild delphinids, are you familiar with the term party?

- Yes
- No

Do you consider party to be a synonym of either of the following terms?

- group
- school
- neither

Please define party

Please list any scientific paper(s) that you use as a basis for your definition of party.

What difficulties have you experienced in applying this definition?

With regards to the study of wild delphinids, are you familiar with the term pod?

- Yes
- No

Do you consider pod to be a synonym of any of the following terms?

- group
- school
- party
- none of the above

Please define pod.

Please list any scientific paper(s) that you use as a basis for your definition of pod.

What difficulties have you experienced in applying this definition?

Please be advised that once you continue you will not be able to change your answers.

Thank you for completing the survey.

If you have any additional comments, please write them below.

**TABLE A1:** Evaluation process used to assess the criteria employed in definitions of delphinid groups

Requirements for each criterion	Criteria	Examples
The definition refers explicitly to…		A group was defined as…
…the distance between the animals or the distance between the animals and some fixed point	Spatial proximity	…all individuals within 10 m of any other member
…the behavior of the animals	Behavior	…all individuals engaged in the same behavior
…the movement and direction of the animals	Movement and directionality	…all individuals that were moving in the same direction
…the number of animals that are present	Number of individuals	…two or more individuals that were…
…the visual range of the observers	Visual range of observers	…all individuals within visual range that were…
…interactions between the animals	Interactions	…any individuals that were interacting
…the time when or the amount of time that the animals are present	Temporal proximity	…individuals that were present at the same time and…
…the species that are present	Species present	…individuals of the same species that were…
